# Supplementary material for: Systematic Investigation of Structural, Morphological, Thermal, Optoelectronic, and Magnetic Properties of High-Purity Hematite/Magnetite Nanoparticles for Optoelectronics
Source: Nanomaterials (Basel). 2022 May 11;12(10):1635. doi: 10.3390/nano12101635 (PMC9144984; doi:10.3390/nano12101635)
Supplement: Supplementary file 1 [file nanomaterials-12-01635-s001.zip › nanomaterials-1686896-supplementary.pdf]

Supplementary Material for:

Systematic Investigation of Structural, Morphological, Thermal, Optoelectronic, and Magnetic Properties of High-Purity Hematite/Magnetite Nanoparticles for Optoelectronics

Akbar Ali Qureshi [1,2], Sofia Javed [1,*], Hafiz Muhammad Asif Javed [3], Muhammad Jamshaid [2], Usman Ali [1] and Muhammad Aftab Akram [1]

1 School of Chemical & Materials Engineering, National University of Sciences & Technology, Islamabad 44000, Pakistan; aali.phdscme@student.nust.edu.pk (A.A.Q.); usman.phdscme@student.nust.edu.pk (U.A.); aftabakram@scme.nust.edu.pk (M.A.A.)
2 Department of Mechanical Engineering, Bahauddin Zakariya University, Multan 60000, Pakistan; muhammad.jamshaid@bzu.edu.pk
3 Department of Physics, University of Agriculture Faisalabad, Faisalabad 38000, Pakistan; m.asif.javed@uaf.edu.pk
* Correspondence: sofia.javed@scme.nust.edu.pk

Supporting Information

Experimental Section

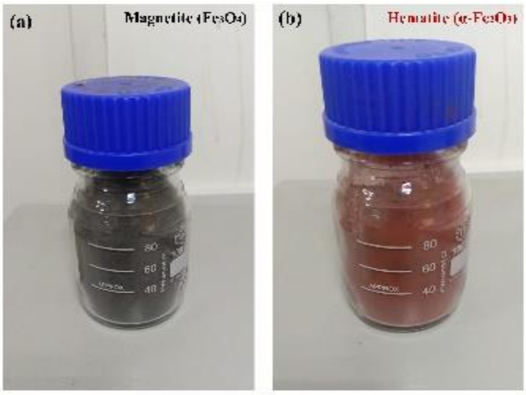

Figure S1. Ball mill samples of (**a**) magnetite (Fe_3O_4) and (**b**) hematite (α-Fe_2O_3) powders

Results Section

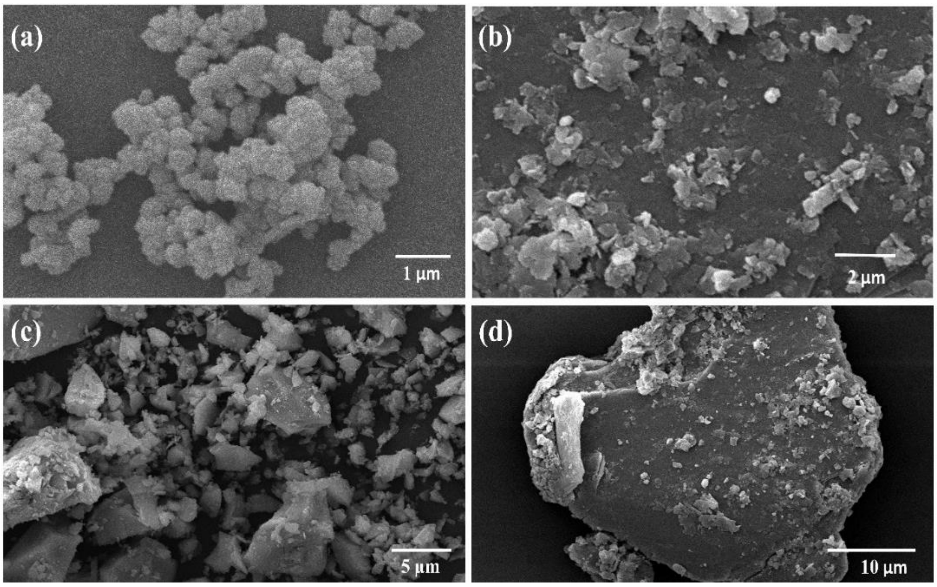

Figure S2. SEM images of the Hematite (α-Fe_2O_3) micro-powder with varying magnifications (**a**) 1 μm, (**b**) 2 μm, (**c**) 5 μm and (**d**) 10 μm before ball mill

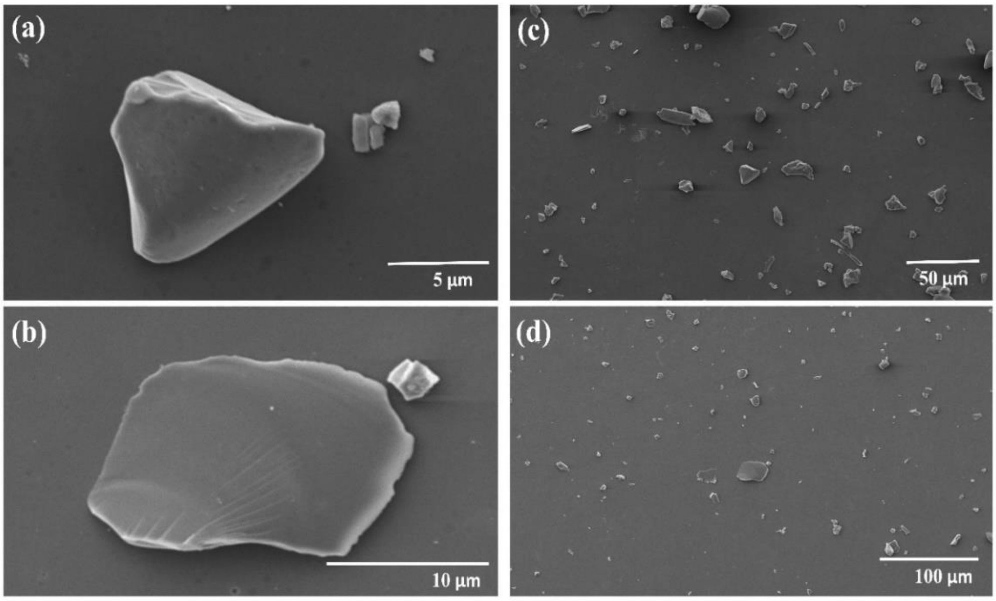

Figure S3. SEM images of the Magnetite (Fe_3O_4) micro-powder with varying magnifications (**a**) 5 μm, (**b**) 10 μm, (**c**) 50 μm and (**d**) 100 μmbefore ball mill

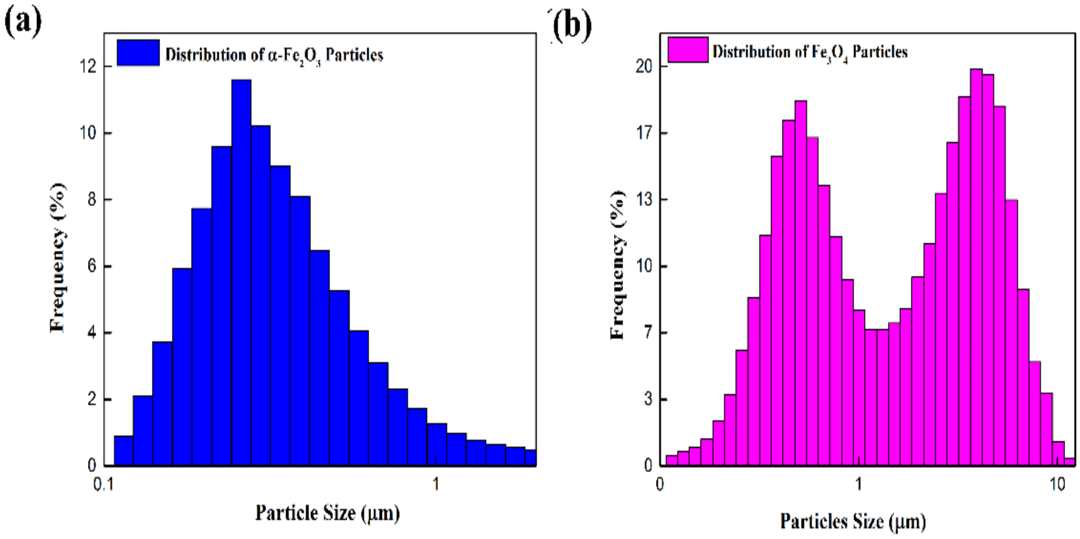

Figure S4. Histogram of (**a**) Hematite (α-Fe_2O_3) and (**b**) Magnetite (Fe_3O_4) micro-powders

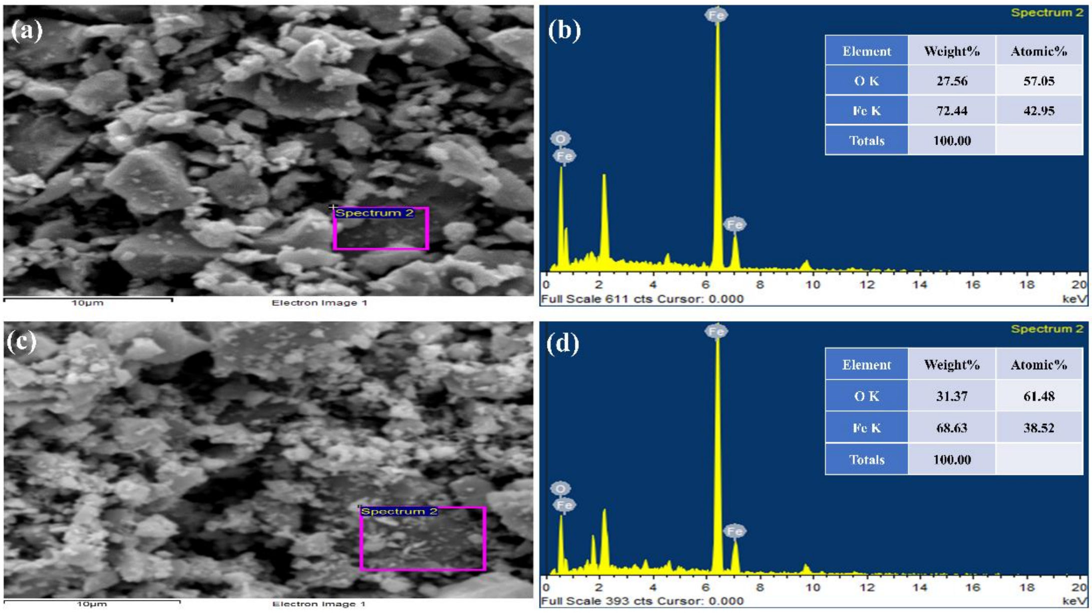

Figure S5. EDX analyses of (**a**), (**b**) Hematite (α-Fe_2O_3) and (**c**), (**d**) Magnetite (Fe_3O_4) Nanoparticles

Table S1. Elemental Composition of Hematite (α-Fe_2O_3) powder by XRF

Element	%
Fe	98.76
Si	0.71
Cr	0.268
Cu	0.153

Nb	0.016
Mo	0.023
Pb	0.069
**Total**	**100**

Table S2. Elemental Composition of Magnetite (Fe_3O_4) powder

**Element**	**%**
Fe	97.29
Si	0.612
S	0.135
Ti	0.47
V	0.424
Cr	0.061
Mn	0.480
Zr	0.208
Nb	0.010
Bi	0.312
**Total**	**100**

The values in Table S1 and Table S2 are only for metallic entities. Oxygen peak is removed during analysis.

Table S3. The calculated crystal size (D) using the Scherrer equation, interplanar spacing d_{hkl}, lattice constants, cell volume, and SEM results

**Sample**	**Empirical formula**	**2θ**	**Plane**	**β**	**D (nm) XRD**	**d_{hkl} (Å)**	**a, b (Å)**	**c (Å)**	**Cell Volume (Å^3)**	**D (nm) SEM**
Hematite	α-Fe_2O_3	33.13	104	0.0984	92	2.70395	5.0370	13.7710	302.58	~85
Magnetite	Fe_3O_4	35.43	311	0.1968	44	2.53296	8.3940	8.3940	591.43	~50

Table S4. Hall Effect Measurements of Hematite (α-Fe_2O_3) and Magnetite (Fe_3O_4) Films

**Sample**	**Composition**	**Bulk Concentration (/cm^3)**	**Mobility (cm^2/Vs)**	**Conductivity (1/Ω.cm)**
Hematite	α-Fe_2O_3	7.79×10^{18}	0.258	1.42×10^{2}
Magnetite	Fe_3O_4	3.92×10^{19}	0.196	1.07×10^{2}

Table S5. Magnetic Properties of Hematite (α-Fe_2O_3) and Magnetite (Fe_3O_4) Nanoparticles

Sample	Composition	M_s *(emu/g)*	H_c *(Oe)*	M_r *(emu/g)*
Hematite	α-Fe_2O_3	1.242	246	0.3
Magnetite	Fe_3O_4	70.34	169.38	8.82
